# Xylazine test strip utilization among pregnant and postpartum women with opioid use disorder: A pilot study

**DOI:** 10.1002/pmf2.70274

**Published:** 2026-03-22

**Authors:** Ilana Hull, Margaret Shang, Elizabeth Krans, Ellen Stewart, Megan Ritter, Lauren Narbey, Samantha Mayo, Raagini Jawa

**Affiliations:** ^1^ Division of General Internal Medicine Department of Medicine University of Pittsburgh Pittsburgh Pennsylvania USA; ^2^ Magee‐Womens Research Institute and Foundation Pittsburgh Pennsylvania USA; ^3^ Department of Obstetrics Gynecology & Reproductive Sciences University of Pittsburgh School of Medicine Pittsburgh Pennsylvania USA; ^4^ Center for Research on Healthcare University of Pittsburgh School of Medicine Pittsburgh Pennsylvania USA; ^5^ Present address: Duquesne University, School of Nursing Pittsburgh Pennsylvania USA

**Keywords:** adulterant, drug checking, fentanyl, harm reduction, opioid use disorder, xylazine

## Abstract

**Introduction:**

The emergence of xylazine, a veterinary alpha‐2 and kappa opioid receptor agonist, as an adulterant in illicitly manufactured fentanyl has created new challenges and risks for pregnant people who use unregulated opioids. Xylazine test strips (XTSs) have been proposed as a harm reduction strategy, but their use has not been studied among pregnant and postpartum populations. The goals of this study were to assess the feasibility, acceptability, and efficacy of a novel Perinatal XTS intervention aimed at reducing xylazine exposure and associated harms.

**Methods:**

Pregnant and postpartum women with opioid use disorder and high risk of ongoing unregulated opioid use were recruited from inpatient units of a high‐volume maternity hospital in western Pennsylvania. Participants engaged in a standardized educational session about xylazine and XTS and then were given five XTS kits. Over a 2‐month period, participants were asked to complete a real‐time survey when they used an XTS to report test results and any associated drug use behavior change. All participants were asked to participate in an end‐of‐study survey and a semi‐structured interview about their experiences with Perinatal XTS.

**Results:**

A total of 50 participants were enrolled, completed the education session, and accepted the XTS kits. Most participants were concerned about the potential impacts of xylazine on *pregnancy* and neonatal outcomes. Seventeen participants submitted a total of 71 real‐time surveys after using XTS to test drug samples. In the majority of cases, a positive result prompted behavioral change. Qualitative interviews with participants revealed six major themes: (1) Xylazine and XTS education was highly acceptable. (2) Desire for additional education related to xylazine. (3) XTS distribution and use were highly acceptable. (4) Impact of XTS on behavior change varied based on participant circumstances. (5) Concerns related to xylazine's impact on maternal health and treatment. (6) Concerns related to xylazine's impact on fetal and neonatal health.

**Conclusion:**

Findings from this pilot study demonstrate that Perinatal XTS, a harm‐reduction intervention combining xylazine and XTS education and XTS kit distribution, is highly feasible to deliver, is acceptable among pregnant and postpartum women cared for in an obstetric healthcare setting, and has the potential to reduce risk‐related behaviors.

## 1 INTRODUCTION

Overdose has become a leading cause of *pregnancy*‐associated death in the United States, particularly driven by the rise of synthetic opioids such as illicitly manufactured fentanyl and increasing rates of polysubstance use [1, 2]. Drug overdose rates in the United States involving illicitly manufactured fentanyl more than tripled from 2016 to 2021, with a 24.1% increase from 2020 to 2021 [3]. The drug crisis has been further complicated by the emergence of xylazine, an adulterant in the illicitly manufactured fentanyl drug supply [4]. The prevalence of xylazine‐adulterated fentanyl has surged in the northeast, especially in Pennsylvania, where xylazine‐involved drug overdose deaths increased by 1000% from 2018 to 2022 [5]. In 2023, the US government designated fentanyl‐xylazine combinations as an “emerging threat,” underscoring the urgent need for research on their impact, including during *pregnancy* [6].

Xylazine, a veterinary sedative acting on alpha‐2 adrenergic and kappa opioid receptors, has never been approved for human use due to severe central nervous system depression observed in early clinical trials [7, 8]. Clinically, xylazine exposure can result in sedation, muscle relaxation, bradycardia, hypotension, dysglycemia, and anemia, and chronic exposure can lead to physiological dependence with a withdrawal syndrome characterized by irritability, anxiety, hypertension, and dysphoria [9]. Xylazine exposure is also associated with the development of severe necrotic skin ulcerations, which can lead to soft tissue infections and in severe cases, amputation [4, 5, 8, 9, 10]. While there are no human studies identifying additional risks of xylazine exposure in *pregnancy*, xylazine does cross the placenta [11]. Animal studies raise concerns for increased myometrial contractility, decreased uterine blood flow, and increased maternal hypoxia and acidosis in the presence of xylazine, which could lead to fetal hypoxia and increase the risk of perinatal complications such as fetal growth restriction, placental insufficiency, and preterm labor [12, 13, 14, 15, 16]. Xylazine has also been detected in the milk of lactating cows, which also raises concerns about infant exposure through breastmilk [17].

Due to the potential for xylazine to exacerbate adverse maternal and neonatal outcomes associated with fentanyl use during *pregnancy*, there is a need for interventions to help pregnant women identify xylazine in the drug supply with the goal of limiting exposure and mitigating risks associated with exposure. A range of drug‐checking methods exists, including single‐use immunoassay test strips that can be used for direct drug testing in the community. The first test strips to be widely distributed in the United States were fentanyl test strips (FTS), with limited available evidence demonstrating high levels of acceptability for FTS as a harm reduction strategy among people who use drugs [18, 19, 20]. There are limited studies that have looked at the impact of FTS use on behavior change but as of 2024, two studies have found that positive FTS results are linked to safer drug use behaviors [19, 21, 22].

In March 2023, xylazine test strips (XTSs) became commercially available, providing a new opportunity to evaluate the impact of immunoassay test strip distribution on drug use behaviors and substance exposures. Despite the potential for XTS to reduce xylazine‐related harms during *pregnancy*, there is currently no evidence supporting their use in perinatal populations who use drugs. The goal of this research was to pilot a novel Perinatal XTS intervention aimed at reducing xylazine exposure and related risks during *pregnancy*. Specifically, our objectives were to: (a) assess the feasibility and acceptability of delivering a harm reduction intervention to pregnant women with unregulated opioid use in acute care settings and (b) explore the intervention's efficacy in influencing drug use behaviors during *pregnancy*.

## 2 METHODS

### 2.1 Study site and participants

From January to September 2024, participants were recruited from inpatient units of a large tertiary care maternity hospital in southwestern Pennsylvania. Eligible participants were 18 years or older, pregnant or within one year postpartum, and reported non‐prescribed opioid use within 3 months prior to enrollment. Recruitment occurred with the assistance of clinical care teams, namely the hospital's perinatal addiction medicine consult team, which provides inpatient substance use treatment, including withdrawal management, initiation of medications for opioid use disorder, and referrals to ongoing treatment. Flyers were also placed in healthcare settings commonly serving pregnant and postpartum patients with unregulated opioid use, including the emergency department and antepartum and labor and delivery units. Eligible individuals completed written informed consent prior to enrollment. After enrollment, the Perinatal XTS intervention was delivered by research staff, and participants were followed for 2 months.

### 2.2 Perinatal XTS intervention

The Perinatal XTS intervention included two components: (a) a standardized educational session on xylazine and XTS use facilitated by non‐clinical research staff and (b) distribution of XTS kits. During the educational session, participants reviewed two pamphlets and watched two short videos on a study tablet. The pamphlets were developed by clinicians at the University of Pittsburgh and Grayken Center for Addiction Training and Technical Assistance (including authors M.S. and R.J.). The first covered xylazine, its potential harms, and overdose prevention strategies [23]. The second reviewed correct XTS use [24]. Participants then viewed two videos: “Xylazine 101,” produced by the Grayken Center (featuring R.J.) [25], and an instructional video from the XTS distributor explaining proper testing procedures [26]. Research staff used the teach‐back method to confirm participants’ understanding of XTS use. The educational session lasted approximately 10–15 min. After completing the education session, the research assistant gave the participant a bag containing five XTS kits, each with two XTS with interpretation instructions, two 5 mL sterile water vials, and two single‐use cookers to use as a sterile mixing surface when completing the testing.

### 2.3 Data collection

Before receiving the intervention, participants completed a baseline survey using REDCap [27, 28] electronic data capture tools hosted at the University of Pittsburgh. The baseline survey assessed demographics, substance use and treatment history, knowledge of xylazine and XTS, and concerns about xylazine exposure during *pregnancy*. The knowledge assessment consisted of 10 True or False questions and a single question that asked participants to identify the potential effects of xylazine exposure. The concern assessment included six questions on a 5‐point Likert scale (1 = *not at all concerned*, 5 = *extremely concerned*) assessing concern about potential effects of xylazine on maternal, fetal, and neonatal health. Both assessments were developed by the study team. After the educational intervention, research staff reviewed the XTS kit contents with participants. Each XTS kit was labeled with a quick‐response (QR) code linking to a brief, real‐time REDCap survey used to report test use, results, and related behavior changes. Participants could submit one real‐time survey per day and up to five during the two‐month follow‐up period. We utilized a QR code to allow individuals without regular access to a phone or email to complete the survey with any available device and included the code on each XTS kit to ensure easy access at the time of XTS use.

At the end of the 2‐month follow‐up period, participants received a final REDCap end‐of‐study survey via text or email to update demographic, substance use, and treatment information. The xylazine knowledge and concerns assessments from enrollment were also re‐administered. Participants were then invited to complete a 15–30 minute semi‐structured interview about their experiences with xylazine in the drug supply, use of XTS as a risk mitigation strategy, and perceptions of the Perinatal XTS intervention. Interviews were conducted by a trained research staff member (E.S.) either by phone or in‐person, audio‐recorded, and transcribed verbatim. Participants could receive up to $175: $50 for the baseline survey, $10 per real‐time XTS survey (up to 5), $25 for the post‐intervention survey, and $50 for the final interview.

### 2.4 Outcomes

Feasibility was evaluated using screening and enrollment data and was defined as successfully enrolling and delivering the Perinatal XTS intervention to more than half of eligible pregnant women approached in healthcare settings. Acceptability was evaluated using XTS kit utilization data and thematic analysis of the qualitative interview responses. Efficacy was evaluated using responses from real‐time XTS use surveys and interview data describing any changes in drug use behaviors following XTS kit use.

### 2.5 Data analysis

Survey data were summarized using descriptive statistics with frequencies and proportions for categorical variables and means and standard deviations for continuous variables. Pre‐ and post‐intervention responses to the XTS knowledge questions were compared using the exact McNemar test for matched pairs. STATA SE Version 18.5 was used to conduct statistical analyses, and statistical significance was defined as <0.05.

Interviews were transcribed verbatim. A preliminary codebook was created based on the interview guide, which focused on eliciting participants’ experiences with xylazine in the drug supply, using XTS as a risk mitigation strategy, and their perspectives on the Perinatal XTS intervention. Two members of the study team (I.H. and L.N.) then coded the first eight interviews inductively based on recurring themes that emerged from the transcripts. Discrepancies between original and emerging codes were reviewed, and the codebook was updated accordingly. The remaining interviews were then coded using the final codebook. Themes were generated using thematic analysis. Dedoose qualitative interview software was used for all coding and analyses. The study was approved by the University of Pittsburgh Institutional Review Board.

## 3 RESULTS

### 3.1 Quantitative

#### 3.1.1 Feasibility and acceptability

From January to September 2024, 67 pregnant and postpartum women with unregulated opioid use presented to the study sites for healthcare services and met eligibility criteria for study participation. Among these, 50 (74.6%) agreed to enroll in the study including 44 (88%) who were pregnant and 6 (12%) who were immediately postpartum at the time of enrollment. Of the 44 pregnant participants, 16(36%) were in the first trimester at enrollment, 17(38%) were in the second trimester, and 11(25%) were in the third trimester. Most participants were approached on the antepartum and postpartum inpatient units. The majority were white (86%), publicly insured (96%), and had an annual household income less than $30,000 (66%, Table 1). Prior use of drug testing strips was reported by 60% of participants, and nearly all (98%) reported utilizing medications for opioid use disorder (MOUD) at some point in their lifetime. A total of 42% were taking methadone, 14% were taking buprenorphine, and 42% were not utilizing MOUD at the time of enrollment, with the MOUD status of one participant at enrollment unknown. Polysubstance use was common among participants, with benzodiazepines (54%) and cocaine (64%) the most frequently reported exposures in addition to opioids (Table 1).

**TABLE 1:** Participants’ demographics, *n* = 50.

	*n*(%)
Mean age, years (SD)	32 (4)
Race	
White only	43 (86)
Black only	2 (4)
Multi‐race	5 (10)
Ethnicity	
Non‐Hispanic	48 (96)
Prefer not to answer	2 (4)
Insurance	
Public	48 (96)
Purchased insurance	1 (2)
No current insurance	1 (2)
Income	
Less than $30,000	33 (66)
$30,000–$60,000	8 (16)
Greater than $60,000	2 (4)
Unsure	7 (14)
Pregnancy status at enrollment	
Pregnant	44 (88)
First trimester	16 (36)^a^
Second trimester	17 (38)^a^
Third trimester	11 (25)^a^
Postpartum	6 (12)
Past year self‐reported substance use	
Heroin or illicitly manufactured fentanyl	48 (96)
Non‐prescribed opioids or painkillers	20 (40)
Benzodiazepines	27 (54)
Cocaine	32 (64)
Methamphetamines	19 (38)
Any MOUD in lifetime	49 (98)
MOUD in lifetime, by type	
Buprenorphine‐naloxone, sublingual	36 (72)
Buprenorphine, subcutaneous injection	7 (14)
Methadone	42 (84)
Naltrexone, injection	12 (24)
Naltrexone, oral	5 (10)
MOUD at enrollment, by type	
Methadone	21 (42)
Buprenorphine	7 (14)
None	21 (42)
Unknown	1 (2)

Abbreviation: MOUD, medications for opioid use disorder.

^a^ Calculated using total number of participants pregnant at enrollment = 44.

**FIGURE 1:**
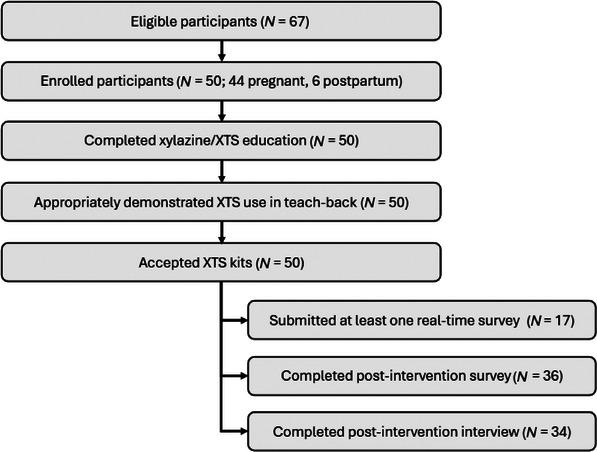
Participants’ eligibility, enrollment, and study completion. XTS, xylazine test strips.

All enrolled participants (100%) completed all aspects of the Perinatal XTS intervention, demonstrated appropriate XTS use post‐education through teach‐back, and accepted the XTS kits. During the 2‐month post‐intervention follow‐up period, 17 (34%) participants submitted at least one real‐time survey after using XTS. Thirty‐six (72%) out of the total 50 participants completed the end‐of‐study surveys, and 34 (68%) participated in a qualitative interview (Figure 1). A total of 16 (44%) of these participants were utilizing buprenorphine at the end of the study and 15 (42%) were utilizing methadone as MOUD.

#### 3.1.2 Xylazine knowledge and concerns

In the baseline survey, most participants (94%) reported at least a general awareness of xylazine. Among the 36 participants who completed both the pre‐ and post‐intervention surveys, overall knowledge about xylazine and the use of XTS stayed the same or improved for over 70% of participants. Participants had a statistically significant improvement in understanding that XTS cannot determine the amount of xylazine present in a drug sample (Table 2).

**TABLE 2:** Xylazine test strip knowledge among study participants who completed both pre‐ and post‐intervention survey, *n* = 36.

XTS questions (correct answers)	Pre‐intervention response*, n*(%)	Post‐intervention responses *n*(%)
Drug‐checking strips are illegal in Pennsylvania (False)	28 (78)	29 (81)
Xylazine test strips (XTSs) are 100% accurate (False)	10 (28)	15 (42)
XTSs can tell you how much xylazine is present (False)	16 (44)	30 (83)*
You can get drug‐checking strips (fentanyl or xylazine) from treatment programs or online (True)	28 (78)	33 (92)

Abbreviation: XTS, xylazine test strips.

* Statistically significant difference with a *p* value of 0.0005.  Statistical significance calculated using exact McNemer test using match pairs for pre‐ and post‐intervention responses for individual participants.

**FIGURE 2:**
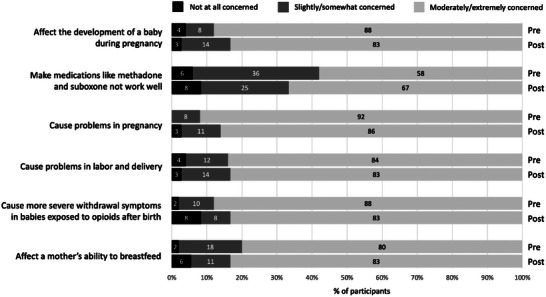
Participants’ concerns about xylazine, pre (*n* = 50) and post (*n* = 36).

The baseline survey also demonstrated that participants were moderately or extremely concerned that xylazine could impact fetal development (88%), exacerbate neonatal withdrawal symptoms (88%), or cause problems in *pregnancy* (92%) or labor and delivery (84%). Over half (58%) of participants were moderately or extremely concerned that xylazine could decrease the effectiveness of MOUD (buprenorphine or methadone). Among the 36 participants who completed both the baseline and post‐intervention surveys, most remained concerned about the potential impacts of xylazine on maternal and neonatal outcomes at the end of the study based on post‐intervention survey responses (Figure 2).

#### 3.1.3 XTS utilization and acceptability

Among 50 enrolled participants, 17 (34%) submitted at least one real‐time XTS survey after testing drug samples. On average, participants submitted 1.56 surveys (SD = 2.17), and 11 participants submitted the maximum of five surveys for a total of 71 real‐time surveys submitted. Most participants used XTS for testing unregulated opioids, although some participants also used XTS to test stimulants. When the XTS were used (*n* = 71), 52% reported a positive xylazine result. In the majority of cases, a positive result prompted a change in drug use behaviors including choosing to not use the tested sample (22%), using less of the drugs than they typically would (51%), changing the route of use (8%), using the drugs with someone else (5%), or purchasing drugs from another source next time (35%) (Table 3).

**TABLE 3:** Changes in drug use behaviors following XTS use, *n* = 71 total real‐time surveys submitted.

Substance tested	
Heroin/fentanyl	62 (87.3%)
Pressed opioid pill	2 (2.8%)
Stimulant	7 (9.9%)
Positive result from XTS	37 (52.1%)
*Behavior Change after positive result* ^a^	
Would not use tested sample	8 (21.6%)
Would use less than they typically would	19 (51.4%)
Change route of use	3 (8.1%)
Use with someone else	2 (5.4%)
Purchase from someone else next time	13 (35.1%)
No change in behavior	5 (13.5%)

Abbreviation: XTS, xylazine test strip.

^a^ Behavior change questions were only asked if XTS result was positive. More than one answer could be selected.

### 3.2 Qualitative

#### 3.2.1 Acceptability, concerns, and remaining gaps in care

Qualitative interviews revealed six major themes that are summarized in Table 4 with illustrative quotes. Participants found the Perinatal XTS education session to be highly acceptable (Theme 1). Most participants found the education session to be helpful and were generally comfortable receiving the information in a healthcare setting. Some expressed a desire for additional guidance—particularly managing xylazine‐related withdrawal and wound care (Theme 2). Participants also found hospital‐based distribution and community use of XTS to be highly acceptable (Theme 3). Participants thought XTS were easy to use, appreciated the rapid results, and generally trusted the results. Many noted feeling empowered and safer by having access to XTS and XTS results.

**TABLE 4:** Perspectives regarding xylazine and XTS among study participants, *n* = 34.

Theme	Subthemes	Representative quotes
1. Xylazine and XTS education was highly acceptable	Appreciated the standardized education provided.	*It was definitely something new to me. It was the first time I've ever heard of anything like that. So, finding out that that's what I was exposed to, it kind of gave me some clarity as to why I struggled so much with getting clean this time on my own*. [Participant 2] *Yes, I knew a lot of it, but I think it was cool for someone who might have heard about Tranq but did not know details about it*. [Participant 23] *It impacted me in a positive way just because, I get to know a little bit… I'm more aware of what I'm actually putting into my body versus not really knowing*. [Participant 49]
	Felt comfortable receiving this education in a healthcare environment.	*Actually it was really comfortable to be able to talk about it with everybody*. [Participant 15] *It was helpful. I mean, I learned, I was educated, I learned something, you know?* [Participant 10]
2. Desire for additional education related to xylazine	Requested more education around xylazine withdrawal	*Maybe just go a little more into details on why you would feel [like that] from xylazine, like you know, what exactly causes you to withdrawal from it*. [Participant 49]
	Requested more education around xylazine wounds and care	*Just more ways to take care of the wounds and more access to resources for that because they're pretty bad. If you let them go like it, it would be very, very dangerous*. [Participant 30]
3. XTS distribution and use were highly acceptable	Were comfortable receiving XTS from acute health care settings	*I didn't feel, like giving me those [test strips] to go home and use, I didn't feel uncomfortable about it. And I think people should do it. And I think it needs…the information needs to be out there*. [Participant 14] *Whenever I was first handed them in the in the hospital, I was a little skeptical. Kind of like are you guys encouraging me to kind of go get drugs to test them? Not like you guys want me to do the drugs. I was just kind of confused on that part, but then it's also like educational. After I thought about it, I was, like you know it's obviously a risk of me coming home and, you know, doing drugs again. So having these would be, you know, just nice to see what's in the drugs. If I did end up having them. So I mean I think it's a good idea*. [Participant 17]
	Found XTS were easy to use and trusted the results	*Yea they were very easy to use. It's pretty simple*. [Participant 3] *Because I could definitely tell from the difference as to what I was using. So yea, definitely, definitely trust the results [of the XTS]*. [Participant 2] *They easy and like… Yes. And very. What is that word? Oh, what is that word? Like they come in handy. *[Participant 10]
	Access to XTS increased feelings of safety and empowerment.	*It made me feel more secure in what I was doing. Knowing that, you know, something that somebody claimed was something that it wasn't. That I was able to verify and see, you know. A couple times people told me that they had no clue—like and I was like ‘well hey, this has xylazine in it’ and they were like ‘oh no it don't’. And I was like ‘oh well it does’. I had to prove it a couple times to people. So it was nice to be able to back up what I was saying*. [Participant 11] *It made me feel safer going through this addiction and trying to navigate my addiction and into my recovery so at least I had the awareness of what I was using. It wasn't so much putting the trust into the drug dealers*. [Participant 2] *It impacted me in a positive way just because, I get to know a little bit… I'm more aware of what I'm actually putting into my body versus not really knowing*. [Participant 49]
4. Impact of XTS on behavior change varied based on participant circumstances	Behavior changed after positive xylazine test.	*If it comes back positive for it, I would normally just not do as much because I know it's going to make me drowsy*. [Participant 49] *If it has it, if it comes up positive, like I won't [use it]. I'll tell all my friends, warn everybody*. [Participant 31] *I did not inject it. Yeah, I've just been snorting. *[Participant 15] *It actually helped me quit using. Or stay away from it. Because I knew how much worse the sickness was. And knowing that it tested positive for it just kind of helps with those urges ironically*. [Participant 2]
	Those with period of abstinence did not use XTS but many shared with others in their community.	*I actually gave them to my friends so, well my associates, or shall I say yeah*. [Participant 27] *I used them one time and then I just never used them again because I never picked up any more bags after that. * [Participant 1]
	XTS have less impact as becomes harder to find supply without it	*It just depends on who had it and who is available and the cheapest. But I think we tried. We tested a couple different kinds and then it was all positive*. [Participant 46] *That [fentanyl with xylazine] is all that's available and people would rather do that than be ill*. [Participant 27]
5. Concerns related to xylazine's impact on maternal health and treatment	Frequent concerns about oversedation and wounds.	*There's so many worries. It literally necrotizes your skin and eats away at it*. [Participant 1] *The overdose is, it can happen so much quicker because when you're using fentanyl, you get a nodding sensation, but when you're using xylazine and fentanyl, it doesn't hit you immediately and then all of a sudden you are passed out, hunched over and you are like a zombie*. [Participant 1]
	Beliefs that xylazine exposure worsened withdrawal symptoms, reducing treatment effectiveness.	*It made it almost impossible to use suboxone to get off of it*. [Participant 2] *Like, just like just like getting sober. It made it that much harder to take the step to get to off the xylazine since there was no withdrawal of medications for that. * [Participant 38] *Yes, a lot. When they mentioned that it could be in what I was using and, cause I went from methadone to try to get on Subutex and it just wasn't working with the withdrawals, and they were having to give me Dilaudid and everything and Clonidine. It just it scared me a bit that it was causing different side effects than I was used to*. [Participant 9]
	Concerns about xylazine effects as a motivation to seek treatment.	*I mean, it was just good to know that this stuff is still in the illicit drug supply, and it doesn't seem to be going anywhere. It's just another reason to stop using. *[Participant 50] *Because I've seen the side effects that xylazine can have on you and you know, how long they can last. You know with people having those sores that you know start eating away at your skin, they don't always pop up where like the injection sites or anything. But I mean, there's people that it still looks like it's it hasn't even healed, and it's been over a year. So yeah, it's a good motivator to want to stop*. [Participant 11]
6. Concerns related to xylazine's impact on fetal and neonatal health	Concerns about neonatal xylazine withdrawal	*I worry about what's gonna happen because I'm pregnant and I was using while pregnant, what the withdrawals are going to look like in my newborn*. [Participant 23]
	Concerns about the impact on fetal development	*One is how it affects my body, and my baby, and her brain and how she's growing and about her health and everything. Yeah, it was kind of worrisome whenever I was doing it. Obviously, I was very worried about my baby*. [Participant 17] *So I worry about like at the time, you know. I was using, I was pregnant. And so I would be worried, you know, like ‘what is this gonna do to my baby.’ Are we gonna have a birth defect because of it or, you know, anything like that. *[Participant 49]

Abbreviation: XTS, xylazine test strip.

Participants also noted that access to XTS and XTS results influenced their decisions and drug use behaviors (Theme 4), facilitating positive behavior change such as using less to reduce the sedating effects of xylazine. Several participants who remained abstinent during the study period reported that since they did not need the XTS for themselves, they distributed XTS kits within their communities to help keep others safe. Several participants did note that the utility of XTS was diminished as the prevalence of xylazine increased in the community, expressing the challenge of finding fentanyl without xylazine. Others also shared that XTSs were less useful in situations where participants were primarily focused on preventing withdrawal symptoms.

Participants also expressed significant concerns about xylazine's effects on maternal, fetal, and neonatal health (Themes 5 and 6), including its potential to worsen neonatal opioid withdrawal syndrome. These concerns motivated some participants to seek treatment; however, many noted that xylazine exposure made withdrawal more severe and existing OUD treatments less effective.

## 4 DISCUSSION

As the drug supply evolves, there is a critical need for interventions that help pregnant populations identify and mitigate risks associated with unintentional exposure to emerging adulterants. This pilot study demonstrates that Perinatal XTS—an intervention combining xylazine education and XTS distribution—is feasible to deliver, well‐accepted among pregnant and postpartum patients in an obstetric setting and may reduce risk‐related behaviors. About 75% of eligible individuals agreed to participate, and all enrolled participants successfully demonstrated proper XTS use and accepted XTS kits, underscoring the feasibility of implementing standardized risk mitigation education in hospital settings.

Education about xylazine and its associated harms was highly acceptable to study participants. Although most were aware of xylazine before the intervention, notable improvements in knowledge about xylazine and XTS use were observed. Interestingly, increased understanding of the unregulated drug supply and its risks appeared to motivate engagement in substance use treatment in addition to adoption of safer use behaviors. Several participants expressed interest in receiving additional education on the adverse effects of chronic xylazine use, including wound care and improved withdrawal management strategies, which identify potential targets of future educational and self‐care interventions for this population.

The high acceptability and influence of XTS use on drug use behaviors among pregnant and postpartum participants parallel findings from previously published data regarding FTS use [18, 19, 20, 21, 22]. Thus, our results extend evidence about the potential benefits of wide distribution of immunoassay test strips to a previously unstudied population. Additionally, secondary distribution of XTS kits by participants who were not using unregulated opioids during the follow‐up period suggests an additional community‐level benefit, analogous to community distribution of naloxone [29]. Participants reflected that objective evidence of xylazine in the unregulated supply, as reflected in positive XTS results, helped explain many of the unwelcome symptoms they had experienced and challenges they were encountering when attempting to engage in treatments that had previously worked well for them. In this way, XTS use not only empowered participants to make informed decisions about their drug use but also prompted behavioral changes aimed at reducing harms from xylazine exposure. These findings are consistent with other published qualitative results from interviews with individuals exposed to xylazine, which demonstrated that fears and dissatisfaction related to the effects of xylazine prompted safer use and treatment‐seeking behaviors [30, 31, 32].

Participants expressed significant concern about the effects of xylazine exposure on *pregnancy*, fetal development, and neonatal outcomes, which motivated XTS use and positive behavioral changes related to substance use and treatment engagement. Many reported that learning about xylazine's prevalence in the local drug supply encouraged them to seek treatment and pursue abstinence during *pregnancy*. However, several participants also noted that xylazine exposure complicated withdrawal experiences and made treatment initiation and adherence more difficult. These findings underscore the need for a comprehensive approach to care in this population that integrates patient education, harm reduction strategies, and multidisciplinary adaptations to withdrawal management and treatment protocols to better support pregnant women who use drugs.

To our knowledge, this study is the first to evaluate the use and impact of any immunoassay drug testing strips among pregnant and postpartum individuals, a population often underrepresented in addiction medicine research. Despite these strengths, several limitations remain. As a pilot study, the small sample size limited statistical analysis and prevented the identification of individual‐level factors predicting XTS use or behavior change. Recruitment exclusively from healthcare settings supports the feasibility of intervention delivery in these environments but restricts generalizability to those not engaged in care. Additionally, only one third of participants completed real‐time surveys after using XTS, which may reflect reduced substance use or redistribution of kits to others, as suggested in qualitative interviews, and thus may not fully represent the overall study population. In addition, to ensure privacy and anonymity when submitting real‐time surveys, which involves self‐reporting possession and use of illegal substances, participants input their study identification number without other confirmation of their identity. It is possible that some of these surveys were completed by those who received XTS through secondary distribution, though we received no indication that this occurred.

## 5 CONCUSION

Our findings indicate that XTS education and distribution has the potential to mitigate harms from unregulated drug use and are feasible to deliver to pregnant and postpartum people in healthcare settings. Given the increase in healthcare utilization during *pregnancy*, hospitalizations offer a critical opportunity to provide interventions such as Perinatal XTS. Expanding access to responsive education and drug‐checking tools in perinatal populations is essential as the drug supply evolves. XTS use may enhance safety and empower pregnant and postpartum people to make informed decisions about drug use and treatment engagement. Continued research is needed to clarify the effects of perinatal xylazine exposure and to refine treatment and withdrawal management protocols for this population.

## CONFLICT OF INTEREST STATEMENT

The authors declare no conflicts of interest.
